# A knowledge elicitation study to inform the development of a consequence model for Arctic ship evacuations: Qualitative and quantitative data

**DOI:** 10.1016/j.dib.2021.107612

**Published:** 2021-11-24

**Authors:** Thomas Browne, Brian Veitch, Rocky Taylor, Jennifer Smith, Doug Smith, Faisal Khan

**Affiliations:** a Faculty of Engineering and Applied Science, Memorial University of Newfoundland, St. John's, Canada; b Ocean, Coastal, and River Engineering Research Centre, National Research Council of Canada, St. John's, Canada; c School of Maritime Studies, Fisheries and Marine Institute of Memorial University of Newfoundland, St. John's, Canada

**Keywords:** Arctic shipping, Expert knowledge, Life-safety, Consequence modelling, Mixed methods design, Semi-structured interviews, Thematic analysis, Survey

## Abstract

Expert knowledge was elicited to develop a life-safety consequence severity model for Arctic ship evacuations (Browne et al., 2021). This paper presents the associated experimental design and data. Through semi-structured interviews, participants identified factors that influence consequence severity. Through a survey, participants evaluated consequence severity of different ship evacuation scenarios. The methodology represents a two-phased mixed methods design. Life-safety consequence severity is measured as the expected number of fatalities resulting from an evacuation. Participants of the study were experts in various fields of the Arctic maritime industry. Sixteen experts participated in the interviews and the survey (sample size: *n* = 16). Sample size for the interviews was based on thematic data saturation. Predominantly the same group of experts participated in the survey. Interviews were analysed using thematic analysis. Interview data informed the development of evacuation scenarios defined in the survey. The interview guide and survey questions are presented. Data tables present the codes that emerged through thematic analysis, including code reference counts and code intersection counts. Data tables present the raw data of participant responses to the survey. This data can support further investigation of factors that influence consequence severity, definition of a broader range of evacuation scenarios, and establishment of associated consequence severities. This data has value to Arctic maritime policy-makers, researchers, and other stakeholders engaged in maritime operational risk management.

**Specifications Table**

**Table:** 

Subject	Ocean and Maritime Engineering
Specific subject area	Consequence modelling of Arctic ship evacuations (expert-based assessment)
Type of data	Tables
How the data were acquired	Semi-structured interviews, followed by a survey. Sixteen experts participated in the interviews and survey (sample size: *n* = 16). Semi-structured interviews were conducted and recorded using Cisco Webex video-conferencing software. QSR Nvivo 1.3 software was used for transcription and thematic analysis of the interview data. Surveys were administered and results collecting using Qualtrics online survey software.
Data format	Processed data: processed data from the semi-structured interviews is provided, including thematic codes and descriptions, code reference counts, and code intersection counts. Raw data: survey data
Description of data collection	Through semi-structured interviews, participants identified factors that influence life-safety consequence severity of Arctic ship evacuations. Interviews were held and recorded using Cisco Webex video-conference software. Interviews were transcribed verbatim and analysed through thematic analysis using QSR Nvivo qualitative analysis software. Interview data informed the development of a survey, in which participants evaluated life-safety consequence severity of different evacuation scenarios. Surveys were administered using Qualtrics online survey software.
Data source location	• Institution: Memorial University of Newfoundland, Ocean Engineering Research Centre • City/Town/Region: St. John's, Newfoundland & Labrador • Country: Canada
Data accessibility	Repository name: Mendeley Data identification number: https://doi.org/10.17632/f4jrwm2tnf.1 Direct URL to data: https://data.mendeley.com/datasets/f4jrwm2tnf/1
Related research article	[1] T. Browne, B. Veitch, R. Taylor, J. Smith, D. Smith, F. Khan; Consequence modelling for Arctic ship evacuations using expert knowledge, Marine Policy, 130 (2021) 104,582. https://doi.org/10.1016/j.marpol.2021.104582.

**Value of the Data**

• These data are important because they provide transparency on established consequence severities for Arctic ship evacuations.
• These data provide a novel contribution to Arctic maritime operational risk management, addressing the lack of ship accident data for Arctic regions which prevents the use of conventional statistical approaches to assess life-safety risk.
• Maritime policy-makers, researchers, ship operators, and other stakeholders engaged in Arctic maritime operational risk management can benefit from these data.
• This data can support further investigation of factors that influence consequence severity, definition of a broader range of evacuation scenarios, and establishment of associated consequence severities for Arctic shipping.

## 1 Data Description

The data for this study is contained in a Microsoft Excel Workbook stored in a Mendeley Data repository (https://doi.org/10.17632/f4jrwm2tnf.1). The Workbook contains twenty-seven separate Worksheets. A description of the data contained in each Worksheet is provided in Table 1.

**Table 1:** Description of the data contained in the Microsoft excel workbook.

Worksheet titles	Data format	Descriptions
1. Code descriptions	Processed	Codes established through thematic analysis of the interview data. Codes are used to categorize segments of text, capturing the meaning of what was said by the participant
2. Code reference count	Processed	The number of times each code was referenced across all interview data.
3. Code intersection matrix	Processed	The number of times each combination of code intersections occurred across all interview data. The same segment of text can fit multiple codes and is referred to as a code intersection.
4. Survey, A1.a ― 8. Survey, A4	Raw	Participant responses to Block A survey questions.
9. Survey, B1 ― 27. Survey, B19	Raw	Participant responses to Block B survey questions.

Acronyms used in the Microsoft Excel Workbook and in this article are defined in Table 2.

**Table 2:** Definition of acronyms.

Acronym	Descriptions
AIRSS	Arctic Ice Regime Shipping System
CCG	Canadian Coast Guard
IRB	Inshore Rescue Boat
NORDREG	Northern Canada Vessel Traffic Services Zone Regulations
NWP	Northwest Passage
POB	Personnel On-board
POLARIS	Polar Operational Limit Assessment Risk Indexing System
SAR	Search and Rescue
SARex	Search and Rescue Exercise
PPE	Personal Protective Equipment
VOO	Vessel of Opportunity

Table 3 provides the interview guide used for the semi-structured interviews.

Table 4 provides the defined Likert scale for level of influence used in the survey.

Table 5 provides the defined Likert scale for likelihood used in the survey.

Table 6 provides the factors and associated levels used to define evacuation scenarios in the survey.

Table 7 provides the ship types and associated numbers of personnel on-board (POB) evaluated for each evacuation scenario in the survey.

Table 8 provides the definitions and indices for life-safety consequence severity used in the survey.

Table 9 provides the Block B evacuation scenarios of the survey.

The Appendix provides the complete survey questionnaire.

## 2 Experimental Design, Materials and Methods

Expert knowledge was elicited through a two-phased mixed methods design [1]. In the first phase, through semi-structured interviews, participants identified factors that influence life-safety consequence severity of Arctic ship evacuations. In the second phase, through a survey, participants evaluated life-safety consequence severity of different evacuation scenarios and the level of influence and likelihood of different factors as they pertain to Arctic ship evacuations and consequence severity. Life-safety consequence severity is measured as the expected number of fatalities resulting from an evacuation [1,2].

Sixteen experts participated in the interviews and survey (sample size: *n* = 16). Sample size for the interviews was based a thematic data saturation [3]. Thematic data saturation is achieved when additional interviews produce no new insights. Thematic data saturation was achieved after thirteen interviews, however a total of sixteen interviews were conducted and included in the data presented here. The process to test for thematic data saturation is described by Browne et al. [1]. Predominantly the same group of experts completed the survey, however three participants left the study after the interviews and three new participants joined for the survey. Details on recruitment and participant backgrounds for both the interviews and survey are provided by Browne et al. [1].

### 2.1 Semi-structured interviews

Through semi-structured interviews, participants identified factors that influence life-safety consequence severity of Arctic ship evacuations. Interviews were conducted and recorded using Cisco Webex video-conference software. Interviews were transcribed verbatim. The semi-structured interview guide is presented in Table 3.

**Table 3:** Interview guide (originally presented by Browne et al. [1]).

**1. Introduction**
1.1 What are some of the challenges of a ship evacuation in Arctic waters, in comparison to non-Arctic waters?

**2. Perceived severity and influencing factors**
2.1 What factors contribute to the potential for loss of life during the evacuation and rescue of a ship in Arctic waters?
2.2 Do certain ship types pose a greater potential for loss of life should evacuation and rescue occur in Arctic waters?
2.3 Does the operational profile of a ship influence the potential for loss of life should evacuation and rescue occur in Arctic waters?
2.4 Are there Arctic regions that pose a greater potential for loss of life should evacuation and rescue occur in Arctic waters?

**3. Closing**
3.1 Considering life-safety for Arctic shipping, what are your biggest concerns?
3.2 Is there anything else you would like to add regarding life-safety for Arctic ships?

QSR Nvivo 1.3 qualitative analysis software was used to conduct thematic analysis of the interview data. The interview data was coded and the most frequently referenced codes and code intersections informed the development of themes. A detailed description of the thematic analysis process is provided by Browne et al. [1].

### 2.2 Survey

The analysed interview data was used to develop the survey. The survey was organized in two blocks, Block A and Block B. The survey was administered using Qualtrix online survey software. The complete survey questionnaire is provided in the Appendix.

#### 2.2.1 Block A description

The level of influence that factors have on response time, evacuee survivability, and the potential for loss of life following an evacuation was evaluated. A five-point Likert scale was used to evaluate level of influence (Table 4). The likelihood of loss of life to occur should an evacuation take place onboard different ship types was evaluated. A five-point Likert scale was used to evaluate likelihood (Table 5).

**Table 4:** Likert scale for level of influence (originally presented by Browne et al. [1]).

1. Extreme influence	2. Major influence	3. Moderate influence	4. Slight influence	5. No influence	6. Prefer not to answer

**Table 5:** Likert scale for likelihood.

1. Extremely likely	2. Very likely	3. Moderately likely	4. Slightly likely	5. Not likely at all	6. Prefer not to answer

#### 2.2.2 Block B description

Participants rated evacuation scenarios for life-safety consequence severity. Factors and ship types used to define evacuation scenarios are presented in Table 6 and Table 7, respectively. Life-safety consequence severity is measured as an expected number of fatalities. The five-point severity scale used to evaluate consequence severity is presented in Table 8. Evacuation scenarios are presented in Table 9.

**Table 6:** Factors used to define evacuation scenarios (originally presented by Browne et al. [1]).

Factors	Levels	
Season	Summer	Winter
Ice conditions	Sea ice	Open water
Wind/sea state	Calm	Severe
Evacuation	Controlled	Uncontrolled
Response time	12 h 24 h	2 days 5 days

**Table 7:** Ship types and POB numbers evaluated for evacuation scenarios (originally presented by Browne et al. [1]).

Ship type	POB
Passenger vessel (e.g. expedition cruise ship)	250
Passenger vessel (e.g. standard cruise ship)	1000
Cargo vessel	25
Fishing vessel	10
Pleasure craft	10

**Table 8:** Life-safety consequence severity definitions (originally presented by Browne et al. [1]; modified from the International Maritime Organization Formal Safety Assessment guidelines [4]).

Severity index	Severity	Effects on human safety	Equivalent fatalities
1	Minor	Single or minor injuries	0.01
2	Severe	Multiple or severe injuries	0.1
3	Significant	Single fatality or multiple severe injuries	1
4	Catastrophic	Multiple fatalities	10
5	Disastrous	Large number of fatalities	100

**Table 9:** Evacuation scenarios (originally presented by Browne et al. [1]).

	Factors				
Scenario	Season	Ice conditions	Wind & sea state	Evacuation	Response time
B1 (Baseline)	Summer	Sea ice present	Calm	Controlled	12 h
B2	Summer	Sea ice present	Calm	Controlled	**24 h**
B3	Summer	Sea ice present	Calm	Controlled	**2 days**
B4	Summer	Sea ice present	Calm	Controlled	**5 days**
B5	Summer	**Open water**	Calm	Controlled	12 h
B6	Summer	Sea ice present	**Severe**	Controlled	12 h
B7	Summer	Sea ice present	Calm	**Rapid/Uncontrolled**	12 h
B8	Summer	**Open water**	**Severe**	Controlled	12 h
B9	Summer	**Open water**	Calm	**Rapid/Uncontrolled**	12 h
B10	Summer	Sea ice present	**Severe**	**Rapid/Uncontrolled**	12 h
B11	Summer	**Open water**	**Severe**	**Rapid/Uncontrolled**	12 h
B12	**Winter**	Sea ice present	Calm	Controlled	12 h
B13	**Winter**	**Open water**	Calm	Controlled	12 h
B14	**Winter**	Sea ice present	**Severe**	Controlled	12 h
B15	**Winter**	Sea ice present	Calm	**Rapid/Uncontrolled**	12 h
B16	**Winter**	**Open water**	**Severe**	Controlled	12 h
B17	**Winter**	**Open water**	Calm	**Rapid/Uncontrolled**	12 h
B18	**Winter**	Sea ice present	**Severe**	**Rapid/Uncontrolled**	12 h
B19	**Winter**	**Open water**	**Severe**	**Rapid/Uncontrolled**	12 h

## Ethics Statements

The experimental design and participant recruitment strategy for this study received ethics review and approval by the Memorial University Interdisciplinary Committee on Ethics in Human Research (ICEHR) and is in compliance with the guidelines of the Tri-Council Policy Statement on Ethical Conduct for Research Involving Humans (ICEHR number 20210767-EN) [1].

## CRediT authorship contribution statement

**Thomas Browne:** Conceptualization, Data curation, Formal analysis, Investigation, Methodology, Writing – original draft, Writing – review & editing. **Brian Veitch:** Funding acquisition, Project administration, Supervision, Methodology, Writing – review & editing. **Rocky Taylor:** Conceptualization, Supervision, Writing – review & editing. **Jennifer Smith:** Formal analysis, Methodology, Writing – review & editing. **Doug Smith:** Supervision, Writing – review & editing. **Faisal Khan:** Funding acquisition, Supervision, Writing – review & editing.

## Declaration of Competing Interest

The authors declare that they have no known competing financial interests or personal relationships that could have appeared to influence the work reported in this paper.

## Appendix Supplementary materials

Image, application 1
