# Supplementary material for: A knowledge elicitation study to inform the development of a consequence model for Arctic ship evacuations: Qualitative and quantitative data
Source: Data Brief. 2021 Nov 24;39:107612. doi: 10.1016/j.dib.2021.107612 (PMC8633861; doi:10.1016/j.dib.2021.107612)
Supplement: Supplementary file 1 [file mmc1.docx]

**Appendix. Survey questionnaire**

**Block A**

| Question A1. **Considering a ship evacuation in Arctic waters, rate the factors for their level of influence on the expected number of fatalities.** | | | | | | |
| --- | --- | --- | --- | --- | --- | --- |
|  | 1. Extreme | 2. Major | 3. Moderate | 4. Slight | 5. No influence | 6. Prefer not to answer |
| Response time |  |  |  |  |  |  |
| No. of POB |  |  |  |  |  |  |
| Passengers onboard |  |  |  |  |  |  |
| Crew experienced in Arctic |  |  |  |  |  |  |
| SAR capacity |  |  |  |  |  |  |
| Proximity to communities |  |  |  |  |  |  |
| Suitability of LSAs |  |  |  |  |  |  |
| Presence of VOO |  |  |  |  |  |  |
| Evacuation in ice |  |  |  |  |  |  |
| Evacuation in open water |  |  |  |  |  |  |
| Weather |  |  |  |  |  |  |
| Temperature |  |  |  |  |  |  |
| Time available to evacuate |  |  |  |  |  |  |
| Ability to communicate |  |  |  |  |  |  |

| Question A2. **Considering a ship evacuation in Arctic waters, rate the factors for their level of influence on response time (i.e. the time from when an emergency alert is sent to when rescue is complete).** | | | | | | |
| --- | --- | --- | --- | --- | --- | --- |
|  | 1. Extreme | 2. Major | 3. Moderate | 4. Slight | 5. No influence | 6. Prefer not to answer |
| Weather |  |  |  |  |  |  |
| Evacuation in ice |  |  |  |  |  |  |
| Evacuation in open water |  |  |  |  |  |  |
| Proximity to SAR resources |  |  |  |  |  |  |
| SAR capacity |  |  |  |  |  |  |
| Presence of VOO |  |  |  |  |  |  |
| Ability to communicate |  |  |  |  |  |  |
| No. of POB |  |  |  |  |  |  |
| Proximity to communities |  |  |  |  |  |  |

| Question A3. **Considering a ship evacuation in Arctic waters, rate the factors for the level of influence they have on the ability of evacuees to survive until rescue.** | | | | | | |
| --- | --- | --- | --- | --- | --- | --- |
|  | 1. Extreme | 2. Major | 3. Moderate | 4. Slight | 5. No influence | 6. Prefer not to answer |
| Temperature |  |  |  |  |  |  |
| Weather |  |  |  |  |  |  |
| Evacuation in ice |  |  |  |  |  |  |
| Evacuation in open water |  |  |  |  |  |  |
| Suitability of LSAs |  |  |  |  |  |  |
| Crew experience in Arctic |  |  |  |  |  |  |
| Time available to evacuate |  |  |  |  |  |  |
| Passengers onboard |  |  |  |  |  |  |

| Question A4. **Considering a ship evacuation in Arctic waters, rate the ship types for the likelihood of a loss of life to occur.** | | | | | | |  |
| --- | --- | --- | --- | --- | --- | --- | --- |
|  | 1. Extremely | 2. Very | 3. Moderately | 4. Slightly | 5. Not likely | 6. Prefer not to answer | |
| Passenger |  |  |  |  |  |  | |
| Cargo |  |  |  |  |  |  | |
| Fishing |  |  |  |  |  |  | |
| Pleasure |  |  |  |  |  |  | |
| Adventure |  |  |  |  |  |  | |

**Block B**

*Instructions*:

You will be asked to evaluate 19 different evacuation scenarios. Scenario factors will be changed systematically.

Life-safety consequence severity refers to the potential level of harm to humans and it is typically narrowed to loss of life. For the following questions, you will be asked to assign the life-safety consequence severity for different evacuation scenarios.

Scenario 1 is the Baseline scenario. For Scenarios 2 - 4, you will be asked to evaluate the influence of response time.

| Question B1. **Considering Scenario 1 (Baseline) described below, rate the level of life-safety consequence severity posed by each of the following vessels.** | | | | | | |  |
| --- | --- | --- | --- | --- | --- | --- | --- |
|  | 1. Minor | 2. Severe | 3. Significant | 4. Catastrophic | 5. Disastrous | 6. Prefer not to answer | |
| Passenger,  250 POB |  |  |  |  |  |  | |
| Passenger, 1000 POB |  |  |  |  |  |  | |
| Cargo, 25 POB |  |  |  |  |  |  | |
| Fishing, 10 POB |  |  |  |  |  |  | |
| Pleasure, 10POB |  |  |  |  |  |  | |
| **Scenario 1 (Baseline)** | | |  |  |  |  | |
| Season | Summer | |  |  |  |  | |
| Ice conditions | Sea ice present | |  |  |  |  | |
| Wind & sea state | Calm | |  |  |  |  | |
| Evacuation | Controlled | |  |  |  |  | |
| Response time | 12 hours | |  |  |  |  | |

| Question B2. **Considering Scenario 2 described below, rate the level of life-safety consequence severity posed by each of the following vessels.** | | | | | | |  |
| --- | --- | --- | --- | --- | --- | --- | --- |
|  | 1. Minor | 2. Severe | 3. Significant | 4. Catastrophic | 5. Disastrous | 6. Prefer not to answer | |
| Passenger,  250 POB |  |  |  |  |  |  | |
| Passenger, 1000 POB |  |  |  |  |  |  | |
| Cargo, 25 POB |  |  |  |  |  |  | |
| Fishing, 10 POB |  |  |  |  |  |  | |
| Pleasure, 10POB |  |  |  |  |  |  | |
| **Scenario 2** | | |  |  |  |  | |
| Season | Summer | |  |  |  |  | |
| Ice conditions | Sea ice present | |  |  |  |  | |
| Wind & sea state | Calm | |  |  |  |  | |
| Evacuation | Controlled | |  |  |  |  | |
| Response time | **24 hours** | |  |  |  |  | |

| Question B3. **Considering Scenario 3 described below, rate the level of life-safety consequence severity posed by each of the following vessels.** | | | | | | |  |
| --- | --- | --- | --- | --- | --- | --- | --- |
|  | 1. Minor | 2. Severe | 3. Significant | 4. Catastrophic | 5. Disastrous | 6. Prefer not to answer | |
| Passenger,  250 POB |  |  |  |  |  |  | |
| Passenger, 1000 POB |  |  |  |  |  |  | |
| Cargo, 25 POB |  |  |  |  |  |  | |
| Fishing, 10 POB |  |  |  |  |  |  | |
| Pleasure, 10POB |  |  |  |  |  |  | |
| **Scenario 3** | | |  |  |  |  | |
| Season | Summer | |  |  |  |  | |
| Ice conditions | Sea ice present | |  |  |  |  | |
| Wind & sea state | Calm | |  |  |  |  | |
| Evacuation | Controlled | |  |  |  |  | |
| Response time | **2 days** | |  |  |  |  | |

| Question B4. **Considering Scenario 4 described below, rate the level of life-safety consequence severity posed by each of the following vessels.** | | | | | | |  |
| --- | --- | --- | --- | --- | --- | --- | --- |
|  | 1. Minor | 2. Severe | 3. Significant | 4. Catastrophic | 5. Disastrous | 6. Prefer not to answer | |
| Passenger,  250 POB |  |  |  |  |  |  | |
| Passenger, 1000 POB |  |  |  |  |  |  | |
| Cargo, 25 POB |  |  |  |  |  |  | |
| Fishing, 10 POB |  |  |  |  |  |  | |
| Pleasure, 10POB |  |  |  |  |  |  | |
| **Scenario 4** | | |  |  |  |  | |
| Season | Summer | |  |  |  |  | |
| Ice conditions | Sea ice present | |  |  |  |  | |
| Wind & sea state | Calm | |  |  |  |  | |
| Evacuation | Controlled | |  |  |  |  | |
| Response time | **5 days** | |  |  |  |  | |

*Instructions*:

For Scenarios 5 - 19, a response time of 12 hours is assumed. The other factors will be changed systematically. Changes compared to the Baseline scenario will be highlighted.

Scenarios 5 - 11 will assess summer evacuation scenarios. Scenarios 12 - 19 will assess winter evacuation scenarios.

**Summer evacuation scenarios (Scenarios 5 - 11)**

| Question B5. **Considering Scenario 5 described below, rate the level of life-safety consequence severity posed by each of the following vessels.** | | | | | | |  |
| --- | --- | --- | --- | --- | --- | --- | --- |
|  | 1. Minor | 2. Severe | 3. Significant | 4. Catastrophic | 5. Disastrous | 6. Prefer not to answer | |
| Passenger,  250 POB |  |  |  |  |  |  | |
| Passenger, 1000 POB |  |  |  |  |  |  | |
| Cargo, 25 POB |  |  |  |  |  |  | |
| Fishing, 10 POB |  |  |  |  |  |  | |
| Pleasure, 10POB |  |  |  |  |  |  | |
| **Scenario 5** | | |  |  |  |  | |
| Season | Summer | |  |  |  |  | |
| Ice conditions | **Open water** | |  |  |  |  | |
| Wind & sea state | Calm | |  |  |  |  | |
| Evacuation | Controlled | |  |  |  |  | |
| Response time | 12 hours | |  |  |  |  | |

| Question B6. **Considering Scenario 6 described below, rate the level of life-safety consequence severity posed by each of the following vessels.** | | | | | | |  |
| --- | --- | --- | --- | --- | --- | --- | --- |
|  | 1. Minor | 2. Severe | 3. Significant | 4. Catastrophic | 5. Disastrous | 6. Prefer not to answer | |
| Passenger,  250 POB |  |  |  |  |  |  | |
| Passenger, 1000 POB |  |  |  |  |  |  | |
| Cargo, 25 POB |  |  |  |  |  |  | |
| Fishing, 10 POB |  |  |  |  |  |  | |
| Pleasure, 10POB |  |  |  |  |  |  | |
| **Scenario 6** | | |  |  |  |  | |
| Season | Summer | |  |  |  |  | |
| Ice conditions | Sea ice present | |  |  |  |  | |
| Wind & sea state | **Severe** | |  |  |  |  | |
| Evacuation | Controlled | |  |  |  |  | |
| Response time | 12 hours | |  |  |  |  | |

| Question B7. **Considering Scenario 7 described below, rate the level of life-safety consequence severity posed by each of the following vessels.** | | | | | | |  |
| --- | --- | --- | --- | --- | --- | --- | --- |
|  | 1. Minor | 2. Severe | 3. Significant | 4. Catastrophic | 5. Disastrous | 6. Prefer not to answer | |
| Passenger,  250 POB |  |  |  |  |  |  | |
| Passenger, 1000 POB |  |  |  |  |  |  | |
| Cargo, 25 POB |  |  |  |  |  |  | |
| Fishing, 10 POB |  |  |  |  |  |  | |
| Pleasure, 10POB |  |  |  |  |  |  | |
| **Scenario 7** | | |  |  |  |  | |
| Season | Summer | |  |  |  |  | |
| Ice conditions | Sea ice present | |  |  |  |  | |
| Wind & sea state | Calm | |  |  |  |  | |
| Evacuation | **Rapid / Uncontrolled** | |  |  |  |  | |
| Response time | 12 hours | |  |  |  |  | |

| Question B8. **Considering Scenario 8 described below, rate the level of life-safety consequence severity posed by each of the following vessels.** | | | | | | |  |
| --- | --- | --- | --- | --- | --- | --- | --- |
|  | 1. Minor | 2. Severe | 3. Significant | 4. Catastrophic | 5. Disastrous | 6. Prefer not to answer | |
| Passenger,  250 POB |  |  |  |  |  |  | |
| Passenger, 1000 POB |  |  |  |  |  |  | |
| Cargo, 25 POB |  |  |  |  |  |  | |
| Fishing, 10 POB |  |  |  |  |  |  | |
| Pleasure, 10POB |  |  |  |  |  |  | |
| **Scenario 8** | | |  |  |  |  | |
| Season | Summer | |  |  |  |  | |
| Ice conditions | **Open water** | |  |  |  |  | |
| Wind & sea state | **Severe** | |  |  |  |  | |
| Evacuation | Controlled | |  |  |  |  | |
| Response time | 12 hours | |  |  |  |  | |

| Question B9 **Considering Scenario 9 described below, rate the level of life-safety consequence severity posed by each of the following vessels.** | | | | | | |  |
| --- | --- | --- | --- | --- | --- | --- | --- |
|  | 1. Minor | 2. Severe | 3. Significant | 4. Catastrophic | 5. Disastrous | 6. Prefer not to answer | |
| Passenger,  250 POB |  |  |  |  |  |  | |
| Passenger, 1000 POB |  |  |  |  |  |  | |
| Cargo, 25 POB |  |  |  |  |  |  | |
| Fishing, 10 POB |  |  |  |  |  |  | |
| Pleasure, 10POB |  |  |  |  |  |  | |
| **Scenario 9** | | |  |  |  |  | |
| Season | Summer | |  |  |  |  | |
| Ice conditions | **Open water** | |  |  |  |  | |
| Wind & sea state | Calm | |  |  |  |  | |
| Evacuation | **Rapid / Uncontrolled** | |  |  |  |  | |
| Response time | 12 hours | |  |  |  |  | |

| Question B10 **Considering Scenario 10 described below, rate the level of life-safety consequence severity posed by each of the following vessels.** | | | | | | |  |
| --- | --- | --- | --- | --- | --- | --- | --- |
|  | 1. Minor | 2. Severe | 3. Significant | 4. Catastrophic | 5. Disastrous | 6. Prefer not to answer | |
| Passenger,  250 POB |  |  |  |  |  |  | |
| Passenger, 1000 POB |  |  |  |  |  |  | |
| Cargo, 25 POB |  |  |  |  |  |  | |
| Fishing, 10 POB |  |  |  |  |  |  | |
| Pleasure, 10POB |  |  |  |  |  |  | |
| **Scenario 10** | | |  |  |  |  | |
| Season | Summer | |  |  |  |  | |
| Ice conditions | Sea ice present | |  |  |  |  | |
| Wind & sea state | **Severe** | |  |  |  |  | |
| Evacuation | **Rapid / Uncontrolled** | |  |  |  |  | |
| Response time | 12 hours | |  |  |  |  | |

| Question B11. **Considering Scenario 11 described below, rate the level of life-safety consequence severity posed by each of the following vessels.** | | | | | | |  |
| --- | --- | --- | --- | --- | --- | --- | --- |
|  | 1. Minor | 2. Severe | 3. Significant | 4. Catastrophic | 5. Disastrous | 6. Prefer not to answer | |
| Passenger,  250 POB |  |  |  |  |  |  | |
| Passenger, 1000 POB |  |  |  |  |  |  | |
| Cargo, 25 POB |  |  |  |  |  |  | |
| Fishing, 10 POB |  |  |  |  |  |  | |
| Pleasure, 10POB |  |  |  |  |  |  | |
| **Scenario 11** | | |  |  |  |  | |
| Season | Summer | |  |  |  |  | |
| Ice conditions | **Open water** | |  |  |  |  | |
| Wind & sea state | **Severe** | |  |  |  |  | |
| Evacuation | **Rapid / Uncontrolled** | |  |  |  |  | |
| Response time | 12 hours | |  |  |  |  | |

**Winter evacuation scenarios (Scenarios 12 - 19)**

| Question B12. **Considering Scenario 12 described below, rate the level of life-safety consequence severity posed by each of the following vessels.** | | | | | | |  |
| --- | --- | --- | --- | --- | --- | --- | --- |
|  | 1. Minor | 2. Severe | 3. Significant | 4. Catastrophic | 5. Disastrous | 6. Prefer not to answer | |
| Passenger,  250 POB |  |  |  |  |  |  | |
| Passenger, 1000 POB |  |  |  |  |  |  | |
| Cargo, 25 POB |  |  |  |  |  |  | |
| Fishing, 10 POB |  |  |  |  |  |  | |
| Pleasure, 10POB |  |  |  |  |  |  | |
| **Scenario 12** | | |  |  |  |  | |
| Season | **Winter** | |  |  |  |  | |
| Ice conditions | Sea ice present | |  |  |  |  | |
| Wind & sea state | Calm | |  |  |  |  | |
| Evacuation | Controlled | |  |  |  |  | |
| Response time | 12 hours | |  |  |  |  | |

| Question B13. **Considering Scenario 13 described below, rate the level of life-safety consequence severity posed by each of the following vessels.** | | | | | | |  |
| --- | --- | --- | --- | --- | --- | --- | --- |
|  | 1. Minor | 2. Severe | 3. Significant | 4. Catastrophic | 5. Disastrous | 6. Prefer not to answer | |
| Passenger,  250 POB |  |  |  |  |  |  | |
| Passenger, 1000 POB |  |  |  |  |  |  | |
| Cargo, 25 POB |  |  |  |  |  |  | |
| Fishing, 10 POB |  |  |  |  |  |  | |
| Pleasure, 10POB |  |  |  |  |  |  | |
| **Scenario 13** | | |  |  |  |  | |
| Season | **Winter** | |  |  |  |  | |
| Ice conditions | **Open water** | |  |  |  |  | |
| Wind & sea state | Calm | |  |  |  |  | |
| Evacuation | Controlled | |  |  |  |  | |
| Response time | 12 hours | |  |  |  |  | |

| Question B14. **Considering Scenario 14 described below, rate the level of life-safety consequence severity posed by each of the following vessels.** | | | | | | |  |
| --- | --- | --- | --- | --- | --- | --- | --- |
|  | 1. Minor | 2. Severe | 3. Significant | 4. Catastrophic | 5. Disastrous | 6. Prefer not to answer | |
| Passenger,  250 POB |  |  |  |  |  |  | |
| Passenger, 1000 POB |  |  |  |  |  |  | |
| Cargo, 25 POB |  |  |  |  |  |  | |
| Fishing, 10 POB |  |  |  |  |  |  | |
| Pleasure, 10POB |  |  |  |  |  |  | |
| **Scenario 14** | | |  |  |  |  | |
| Season | **Winter** | |  |  |  |  | |
| Ice conditions | Sea ice present | |  |  |  |  | |
| Wind & sea state | **Severe** | |  |  |  |  | |
| Evacuation | Controlled | |  |  |  |  | |
| Response time | 12 hours | |  |  |  |  | |

| Question B15. **Considering Scenario 15 described below, rate the level of life-safety consequence severity posed by each of the following vessels.** | | | | | | |  |
| --- | --- | --- | --- | --- | --- | --- | --- |
|  | 1. Minor | 2. Severe | 3. Significant | 4. Catastrophic | 5. Disastrous | 6. Prefer not to answer | |
| Passenger,  250 POB |  |  |  |  |  |  | |
| Passenger, 1000 POB |  |  |  |  |  |  | |
| Cargo, 25 POB |  |  |  |  |  |  | |
| Fishing, 10 POB |  |  |  |  |  |  | |
| Pleasure, 10POB |  |  |  |  |  |  | |
| **Scenario 15** | | |  |  |  |  | |
| Season | **Winter** | |  |  |  |  | |
| Ice conditions | Sea ice present | |  |  |  |  | |
| Wind & sea state | Calm | |  |  |  |  | |
| Evacuation | **Rapid / Uncontrolled** | |  |  |  |  | |
| Response time | 12 hours | |  |  |  |  | |

| Question B16. **Considering Scenario 16 described below, rate the level of life-safety consequence severity posed by each of the following vessels.** | | | | | | |  |
| --- | --- | --- | --- | --- | --- | --- | --- |
|  | 1. Minor | 2. Severe | 3. Significant | 4. Catastrophic | 5. Disastrous | 6. Prefer not to answer | |
| Passenger,  250 POB |  |  |  |  |  |  | |
| Passenger, 1000 POB |  |  |  |  |  |  | |
| Cargo, 25 POB |  |  |  |  |  |  | |
| Fishing, 10 POB |  |  |  |  |  |  | |
| Pleasure, 10POB |  |  |  |  |  |  | |
| **Scenario 16** | | |  |  |  |  | |
| Season | **Winter** | |  |  |  |  | |
| Ice conditions | **Open water** | |  |  |  |  | |
| Wind & sea state | **Severe** | |  |  |  |  | |
| Evacuation | Controlled | |  |  |  |  | |
| Response time | 12 hours | |  |  |  |  | |

| Question B17. **Considering Scenario 17 described below, rate the level of life-safety consequence severity posed by each of the following vessels.** | | | | | | |  |
| --- | --- | --- | --- | --- | --- | --- | --- |
|  | 1. Minor | 2. Severe | 3. Significant | 4. Catastrophic | 5. Disastrous | 6. Prefer not to answer | |
| Passenger,  250 POB |  |  |  |  |  |  | |
| Passenger, 1000 POB |  |  |  |  |  |  | |
| Cargo, 25 POB |  |  |  |  |  |  | |
| Fishing, 10 POB |  |  |  |  |  |  | |
| Pleasure, 10POB |  |  |  |  |  |  | |
| **Scenario 17** | | |  |  |  |  | |
| Season | **Winter** | |  |  |  |  | |
| Ice conditions | **Open water** | |  |  |  |  | |
| Wind & sea state | Calm | |  |  |  |  | |
| Evacuation | **Rapid / Uncontrolled** | |  |  |  |  | |
| Response time | 12 hours | |  |  |  |  | |

| Question B18. **Considering Scenario 18 described below, rate the level of life-safety consequence severity posed by each of the following vessels.** | | | | | | |  |
| --- | --- | --- | --- | --- | --- | --- | --- |
|  | 1. Minor | 2. Severe | 3. Significant | 4. Catastrophic | 5. Disastrous | 6. Prefer not to answer | |
| Passenger,  250 POB |  |  |  |  |  |  | |
| Passenger, 1000 POB |  |  |  |  |  |  | |
| Cargo, 25 POB |  |  |  |  |  |  | |
| Fishing, 10 POB |  |  |  |  |  |  | |
| Pleasure, 10POB |  |  |  |  |  |  | |
| **Scenario 18** | | |  |  |  |  | |
| Season | **Winter** | |  |  |  |  | |
| Ice conditions | Sea ice present | |  |  |  |  | |
| Wind & sea state | **Severe** | |  |  |  |  | |
| Evacuation | **Rapid / Uncontrolled** | |  |  |  |  | |
| Response time | 12 hours | |  |  |  |  | |

| Question B19. **Considering Scenario 19 described below, rate the level of life-safety consequence severity posed by each of the following vessels.** | | | | | | |  |
| --- | --- | --- | --- | --- | --- | --- | --- |
|  | 1. Minor | 2. Severe | 3. Significant | 4. Catastrophic | 5. Disastrous | 6. Prefer not to answer | |
| Passenger,  250 POB |  |  |  |  |  |  | |
| Passenger, 1000 POB |  |  |  |  |  |  | |
| Cargo, 25 POB |  |  |  |  |  |  | |
| Fishing, 10 POB |  |  |  |  |  |  | |
| Pleasure, 10POB |  |  |  |  |  |  | |
| **Scenario 19** | | |  |  |  |  | |
| Season | **Winter** | |  |  |  |  | |
| Ice conditions | **Open water** | |  |  |  |  | |
| Wind & sea state | **Severe** | |  |  |  |  | |
| Evacuation | **Rapid / Uncontrolled** | |  |  |  |  | |
| Response time | 12 hours | |  |  |  |  | |
